# Safety assessments of subcutaneous doses of aragonite calcium carbonate nanocrystals in rats

**DOI:** 10.1007/s11051-017-3849-z

**Published:** 2017-05-11

**Authors:** Alhaji Zubair Jaji, Zuki Abu Bakar Zakaria, Rozi Mahmud, Mohamad Yusof Loqman, Mohamad Noor Mohamad Hezmee, Yusuf Abba, Tijani Isa, Saffanah Khuder Mahmood

**Affiliations:** 1 Department of Veterinary Preclinical Science, Faculty of Veterinary Medicine, Universiti Putra Malaysia, Serdang, Malaysia; 2 Department of Veterinary Anatomy, Faculty of Veterinary Medicine, University of Ilorin, Ilorin, Nigeria; 3 Institute of Bioscience, Universiti Putra Malaysia, 43400 Serdang, Selangor Malaysia; 4 Department of Imaging, Faculty of Medicine & Health Science, Universiti Putra Malaysia, Serdang, Malaysia; 5 Department of Companion Animal Medicine and Surgery, Faculty of Veterinary Medicine, Universiti Putra Malaysia, Serdang, Malaysia; 6 Department of Veterinary Pathology and Microbiology, Faculty of Veterinary Medicine, Universiti Putra Malaysia, Serdang, Malaysia

**Keywords:** CaCO_3_, In vivo, Nanotoxicity, Cockle shell, Aragonite

## Abstract

Calcium carbonate nanoparticles have shown promising potentials in the delivery of drugs and metabolites. There is however, a paucity of information on the safety of their intentional or accidental over exposures to biological systems and general health safety. To this end, this study aims at documenting information on the safety of subcutaneous doses of biogenic nanocrystals of aragonite polymorph of calcium carbonate derived from cockle shells (ANC) in Sprague-Dawley (SD) rats. ANC was synthesized using the top-down method, characterized using the transmission electron microscopy and field emission scanning electron microscope and its acute and repeated dose 28-day trial toxicities were evaluated in SD rats. The results showed that the homogenous 30 ± 5 nm-sized spherical pure aragonite nanocrystals were not associated with mortality in the rats. Severe clinical signs and gross and histopathological lesions, indicating organ toxicities, were recorded in the acute toxicity (29,500 mg/m^2^) group and the high dose (5900 mg/m^2^) group of the repeated dose 28-day trial. However, the medium- (590 mg/m^2^ body weight) and low (59 mg/m^2^)-dose groups showed moderate to mild lesions. The relatively mild lesions observed in the low toxicity dosage group marked the safety margin of ANC in SD rats. It was concluded from this study that the toxicity of CaCO_3_ was dependent on the particulate size (30 ± 5 nm) and concentration and the route of administration used.

## Introduction

Cockle (*Anadara granosa*) is a group of generally small, edible, saltwater clams, marine bivalve molluscs of the family Cardiidae shell. Cockle is by far the most vital species cultured, and one of the most common sources of calcium carbonate found in Malaysia. It easily fulfils the increasing demand of biomaterials due to its low cost and availability (Combes et al. 2006; Hoque et al. 2013). The cockle shells contain more than 98% calcium carbonate and thus have the potential to be a starting material for the development of biomaterials for orthopaedic applications (Awang-Hazmi et al. 2007).

The aragonite polymorph of calcium carbonate is a less thermodynamically stable and a less available form of crystalline calcium carbonate polymorph synthesized in the laboratory. The size and shape of aragonite are strongly dependent on the preparation methods and conditions (Wang et al. 1999). Due to the huge striking properties of the aragonite nanoparticles as a material of biomedical importance, researchers have paid huge attention on invention of methods for its synthesis and usage (Guo et al. 2007; Wang et al. 2006).

Nanoparticles (NPs) are nanoobjects with all external dimensions in the nanoscale, where the lengths of the longest and the shortest axes of the nanoobject do not differ significantly (ISO/TS 2015). NPs have properties that are quite unique from their sourced bulk materials. Their sizes are inversely proportional to their surface/volume ratio and chemical reactivity; this makes them interesting materials in research and applications. Thus, significantly improving many fields of human endeavours (Gwinn and Vallyathan 2006; Hristozov and Malsch 2009; Morose 2010; Moorthi et al. 2011). Though renowned with numerous benefits, there are still growing concerns that deliberate or accidental human exposures to some types of NPs, through environmental contamination and distorted ecosystem, may lead to significant adverse health effects (Colvin 2003; Oberdorster et al. 2005; The Royal Society 2004). The fact that the potentials of exposure to NPs are bound to increase, just as their usage, raises pertinent concerns about their health safety (Drobne 2007). These concerns lead to the emergence of a new branch of research in toxicology called nanotoxicology. Toxicology is the study of sequence of events associated with the acquaintance, progress, distribution, metabolism and culminating in cellular macromolecular, DNA or proteins, interactions and the associated toxic manifestations of poisons (Hodgson 2010). Nanotoxicology aims at (i) studying the properties of nanomaterials in toxicity studies; (ii) studying the possible detrimental effects of exposures to NPs; and (iii) recommending comprehensive test protocols for in human and environmental risk assessment of nanomaterials (Oberdorster et al. 2005; Drobne 2007; Nel et al. 2006).

With the recent advancement in the use of NPs in drug delivery systems, there is an urgent need for their risk assessment. Risk assessment involves data collection, analysis and interpretation on the risk of a given entity. Evaluations of dose and hazard of a chemical substance mark the first line of action in its risk assessment. However, such assessments are often strongly complicated by the size and surface dependent behaviour of the tested substances (Elsaesser and Howard 2012). Time- or incident-dependent changes for exposure of NPs in the system have been observed to be best for the evaluation of systemic biology. The interactions and relations following such exposures are often described in biological pathways and networks as preludes to systemic study of nanotoxicity (Kitano 2002).

The respiratory, integumentary and digestive systems have been identified as the three main entry routes of NPs into the body (Stern and McNeil 2008). The fact that NPs could gain access to, and accumulate in, other organs through blood, by biodistribution and bioaccumulation, poses major concerns (Borm et al. 2006; Sayes and Warheit 2009). Apart from blood, phagocytosis and endocytosis of NPs by body cell have also been observed to play very important roles in further spread to distant organs (Garnett and Kallinteri 2006; Yacobi et al. 2010; Greulich et al. 2011). Irrespective of natural barriers, low concentrations of NPs have been found in the liver, the spleen, the heart and the brain (Ji et al. 2006; Oberdorster et al. 2002). There are unanswered queries on the fate of NPs and their residues in the body or whether they accumulate in certain organs. The full mechanisms behind certain in vivo toxicological findings need to be elucidated. For instance, the mechanism of their excretion through the urine is still unclear and there is a need for assessing their possible roles in the blockage of the excretory systems. (Elsaesser and Howard 2012).

The continuous assemblage of engineered NPs as drug carrier systems stresses the need for a full understanding of their health safety (Kroll et al. 2012). Though calcium carbonate is regarded as being generally safe and is now gaining acceptance as a successful nanocarriers for subcutaneous delivery of biologicals (Ueno et al. 2004; He et al. 2008; Higaki et al. 2006), there is paucity of information on the possible toxicity that may arise from deliberate or accidental exposure to its high doses. This study aims at evaluating the acute and subchronic toxicity of subcutaneous doses of cockle shell-derived aragonite calcium carbonate nanocrystals (ANC) in male and female SD rats, with the view of documenting information on its health safety.

## Materials and methods

### Preparation of spherical shaped ANC

This study adopted and modified the Islam et al. (2012) top-down method of nanoparticle production in syntheses of ANC from cockle shells, towards improving its biocompatibility. Micron aragonite calcium carbonate powder was first prepared from cockle shells. This entailed washing and scrubbing of dirt and tissues off the cockle shells. The cleaned shells were boiled at 100 °C for 10 min in HPLC-grade water (resistance >18 M/cm), produced by a Milli-RO6 plus Milli-Q Water System (Organex) and later cooled to room temperature. A second thorough washing with distilled water was also done before oven drying the shells in Memmert UM500 oven (GmbH Co, Germany) at 50 °C for 7 days. The shells were then powdered finely with mortar and pestle (Agate Top diameter 90 mm), ground with a stainless steel blender (Blendor, HCB 550, USA) and sifted using a 75-μm aperture sized stainless steel laboratory test sieve (Endecott Ltd., London, England) to get a 75-μm diameter sized particles. The coarse unfiltered remnants were further dried in the oven for 10 h, and ground with mortar and pestle and blender and sieved to further reduce their diameter. The produced micron aragonite CaCO_3_ powder (MAC) was further desiccated in an oven at 50 °C for 7-day duration for complete dry up. The MAC was then packaged in a Jp Packaging polyethylene plastic bag (Jp Packaging (M) Sdn Bhd).

A measure of 2 g of the 75 μm-sized powder was placed in a 100-mL flat bottom flask, 50 mL of HPLC-grade water (resistance >18 M cm), produced by a Milli-RO6 plus Milli-Q Water System (Organex) and 0.5 mL of dodecyl dimethyl betaine (BS12) (Sigma Aldrich) were added to each flask and stirred vigorously at 1000 rpm, in room temperature, for 90 min using a Systematic Multi-Hotplate Stirrer (DH.WMH03506 DAIHAN WiseStir® SMHS Systematic Multi-Hotplate Stirrers, 3 ^a^ 2 Places 6 Positions, Korean) and a magnetic stirrer bar. The slurry that was obtained from this process was filtered and rinsed with 18.0 cm sized double ring filter papers (Filtres Fioroni, China). The final products was dried in the Memmert UM500 Oven, GmbH Co, Germany) for 24 h at 100 °C and packed in JP packaging polyethylene plastic bags and stored in moisture free enclosure (at 50^o^C) for further analysis and usage.

### Characterization of ANC

The transmission electron microscope (TEM) (Hitachi H-7100, Japan) was used to determine the shape and size of ANC. Sample preparation for TEM entailed dispersal of 100 μg of ANC powder in 1 mL of 100% acetone. This was followed by ultra-sonication (Power Sonic 505, S. Korea) for 30 min. A drop of the supernatant was then placed onto a carbon-covered copper grids placed on a filter paper and left to dry at room temperature. The TEM measurement was made at 150 kV.

The field emission scanning electron microscope (FESEM) (JEOL 7600F, JEOL, München, Germany) GmbH was used to further evaluate the shape and size of ANC. Samples for FESEM analysis were sparingly dispersed on adhesive-coated metallic stub that was then fitted in to the FESEM microscope for the analysis.

ANC was suspended in deionized water and its mean zeta potential was based on dynamic light scattering determined by photon correlation spectroscopy using Zetasizer (Ver. 6.12, serial number MAL1042820, Malvern Instruments Ltd.).

Detailed physicochemical characterization and cytocompatibility of ANC had earlier been determined (Jaji et al. 2017).

### In vivo toxicity of ANC

The protocols for these studies were approved by the Institutional Animal Care and Use Committee (IACUC), Universiti Putra Malaysia (AUP number R002/2014). Rats were procured, from the Animal Resource Unit, University Putra Malaysia (UPM), and kept in the animals housing facility, Faculty of Veterinary Medicine, UPM. They were housed and maintained at constant temperature, with 12 h light/12 h dark cycles and provided with commercial feed (gold coin mouse pellet) and water ad libitum. The handling of the animals was in adherence with the approved IACUC guidelines.

#### Fourteen-day acute toxicity study

Two consecutive sets of apparently normal 10-week-old female SD rats, two groups (toxicity and control groups) per set (three rats per group), were procured and used for preliminary evaluations of acute toxicity of ANC at two different subcutaneous doses (1770 and 11,800 mg/m^2^). In the absence of mortality from the two dose trials (14 days per trial), a last set of rats with similar features as the previous ones was administered single subcutaneous doses of 29,500 mg/m^2^ and closely monitored for signs of toxicity and mortality for 14 days.

#### Repeated dose 28-day trial

Four groups, composing of six apparently normal (three males and three females) 6-week-old SD rats, were procured and used for this evaluation. The animals were divided into three different concentrations groups low, medium and high, and were administered daily subcutaneous doses of 59, 590 and 5900 mg/m^2^ of ANC, respectively. An equivalent volume of vehicle was administered to a control group. The animals were closely monitored for signs of toxicity and mortality for 28 days.

#### Tissues processing

Bones were fixed in 10% neutral formalin for 3 days, during which the formalin was changed every 24 h. The bones were transferred to 80% formic acid for decalcification for 1 week. Decalcified bones and 2 cm visceral specimens were fixed in 10% neutral formalin for 3 days for histological slides production. The tissues were then embedded in paraffin and sectioned at 5 μm thickness. These sections were transferred onto slides and stained with haematoxylin and eosin (Muhammad et al. 2013). The sections were later observed and captured using the Motic Compound Microscope BA410. The Motic Images Plus 2.0 software was used to analyse the images before they were being captured.

### Statistical analyses

Descriptive (mean and standard deviation (SD) and analytical (*t* test and one-way ANOVA with Tukey’s post test) statistics were performed using GraphPad Prism version 5.00 for Windows, GraphPad Software, San Diego, CA, USA. Means of trial doses and control groups were compared at 95% level of significance.

## Results

### Characterization of ANC

**Fig. 1:**
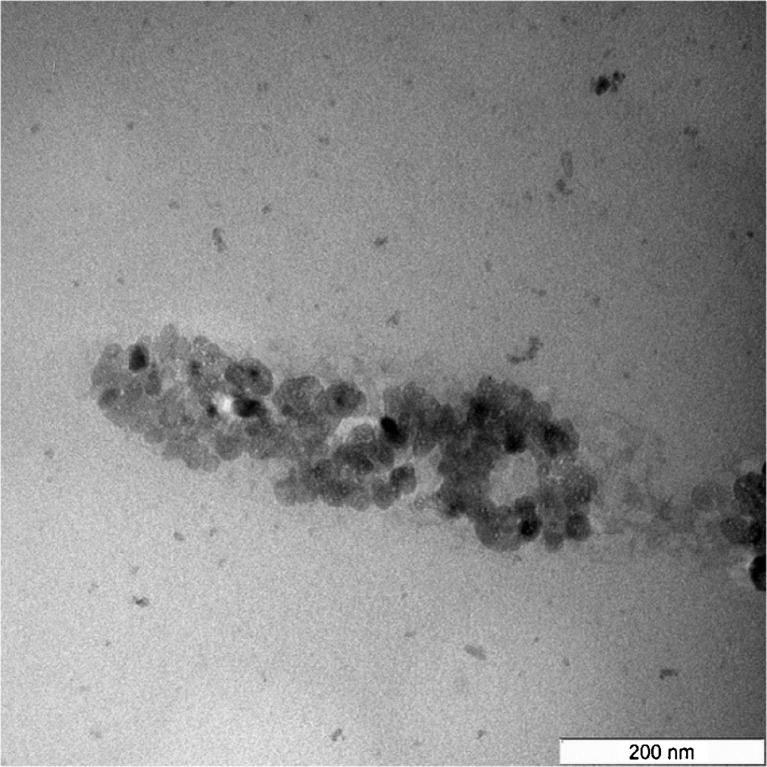
TEM micrograph of the clear spherical crystals of ANC

**Fig. 2:**
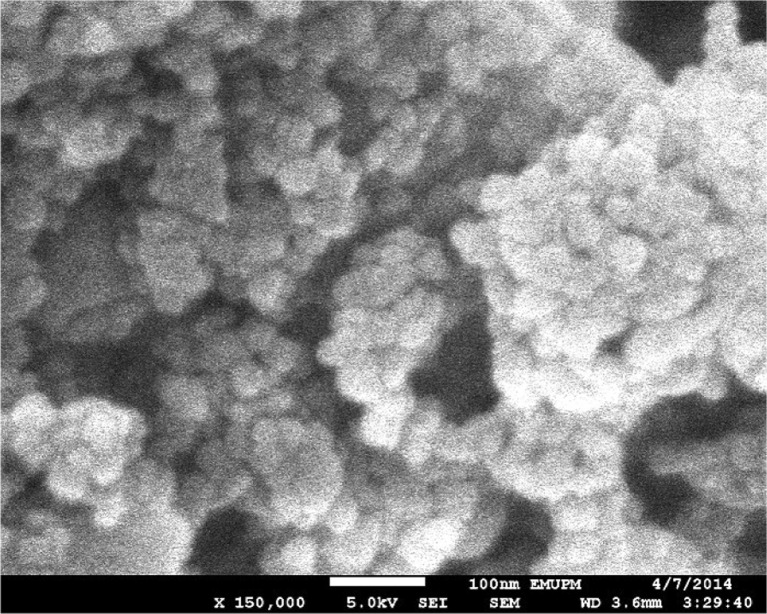
FESEM micrograph of the clear spherical crystals of ANC

**Fig. 3:**
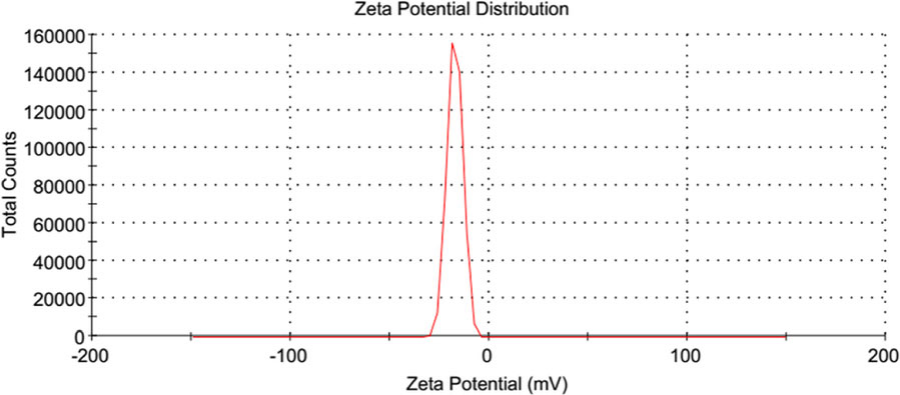
Zeta potential of ANC (−17.2 mV)

Figures 1 and 2 show the shape and size of ANC as viewed under TEM and FESEM, respectively. The crystals were observed to be spherical in shape and the sizes were 30 ± 5 nm in diameter. The zeta potential of ANC was −17.2 mV (Fig. 3).

### Fourteen-day acute toxicity study

#### Clinical observations

**Fig. 4:**
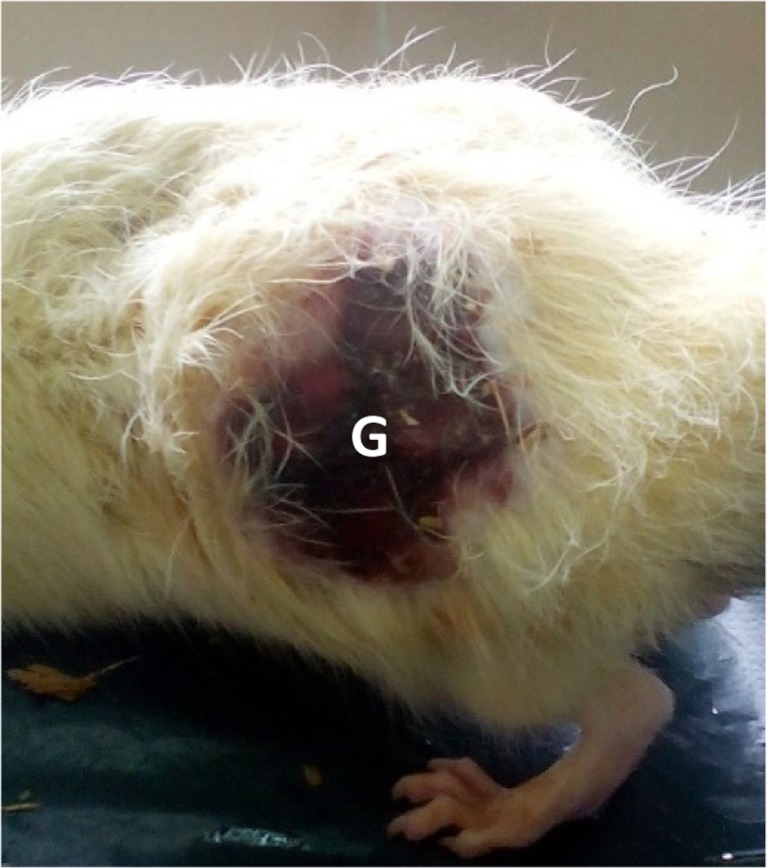
Gross lesion from 14-day acute toxicity study of 29,500 mg/m^2^ ANC. Note the roughened hair coat and gangrened tissue, *G* (blackish discoloration), at the site of injection in one of the rats at day 13 post injection

No mortality was recorded at the end of the experiment. There was no sign of toxicity from the initial doses of the experiment (1770 and 11,800 mg/m^2^). However, the dose (29,500 mg/m^2^) that was eventually used for the study was associated with some toxic signs. The toxicity group showed oedema at the site of injection right from day 3 of the experiment. The rats became anorexic lethargic and dyspnoeic on the sixth day of the experiment. There were also fever, tachycardia and roughened hair coat. These signs advanced in severity in the remaining days of the experiment. The oedema at the site of injection subsided and was replaced by necrosis. The rats showed a serious gangrene lesion at the end of the experiment (Fig. 4).

#### Haematology and serum biochemistry

Some of the haematological parameters in the toxicity group showed insignificant variations from the control. However, serum biochemistry in the toxicity group showed significant increases in alanine aminotransferase (ALT) (*p* < 0.001), alkaline phosphatase (ALP) (*p* < 0.05), aspartate aminotransferase (AST) (0.001) total bilirubin (*p* < 0.001), creatinine, urea (*p* < 0.001), total protein (*p* < 0.001) and potassium (*p* < 0.05). On the contrary, significant decreases were observed in the levels of calcium (*p* < 0.001), cholesterol (*p* < 0.05) and albumin. Increased levels of ALT, ALP, AST and bilirubin signified liver disease while increased levels of urea and creatinine signified kidney disease. Decreased level of albumin was observed to be due to the inability of the diseased liver to produce albumin. This led to compromised integrity of capillaries and congestion and oedema in tissues. Blood haemogram showed a significant decrease in levels of red blood cells (*p* < 0.001), haemoglobin (*p* < 0.001), packed cell volume (haematocrit) (*p* < 0.01), MCHC (*p* < 0.05) and thrombocytes (*p* < 0.05) all signifying anaemia. The anaemia was further characterized with compensatory increased levels of MCV (*p* < 0.05) and white blood cells (*p* < 0.01). There was also significant eosinophilia (*p* < 0.01) in effort to contain the systemic spread of ANC (Tables 1 and 2).

**Table 1:** Serum biochemistry of rats in 14-day acute toxicity of 29,500 mg/m^2^ ANC (mean ± SD)

Parameter	Unit	Control			Toxicity		
		Mean		SD	Mean		SD
Calcium	mmol/L	2.77	±	0.04	2.38	±	0.02**
Phosphate	mmol/L	2.32	±	0.15	2.54	±	0.01
Alanine aminotransferase	U/L	49.37	±	2.22	72.17	±	3.35**
Alkaline phosphatase	U/L	221.30	±	29.14	282.00	±	13.53*
Aspartate aminotransferase	U/L	78.53	±	6.77	139.60	±	5.97**
Total bilirubin	umol/L	2.07	±	0.15	3.07	±	0.15**
Cholesterol	mmol/L	5.85	±	0.32	4.58	±	0.47*
Creatinine	umol/L	34.67	±	3.79	66.33	±	2.08**
Glucose	mmol/L	5.90	±	0.70	5.97	±	0.45
Urea	mmol/L	17.53	±	0.78	27.87	±	0.86**
Total protein	g/L	61.30	±	1.21	71.83	±	1.60**
Albumin	g/L	40.87	±	3.92	29.63	±	2.70*
Sodium	mmol/L	144.30	±	6.81	136.00	±	3.00
Potassium	mmol/L	3.13	±	0.21	4.17	±	0.60*
Chloride	mmol/L	104.00	±	3.61	99.67	±	0.58

*n* = 3 rats per group

*Significant (*p* < 0.05); **significant (*p* < 0.01)

**Table 2:** Haemogram of rats in 14-day acute toxicity of 29,500 mg/m^2^ ANC (mean ± SD)

Parameter	Unit	Control			Toxicity		
		Mean		SD	Mean		SD
Red blood cells	×10^12^/L	8.62	±	0.13	7.06	±	0.09***
Haemoglobin	g/L	157.00	±	1.00	133.40	±	4.05***
Packed cell volume	L/L	0.46	±	0.02	0.36	±	0.02**
MCV	fL	45.55	±	3.21	54.89	±	3.75*
MCHC	g/L	367.30	±	8.05	339.10	±	10.39 *
White blood cells	×10^9^/L	7.21	±	0.58	12.69	±	0.95**
Neutrophils	×10^3^/μL	0.66	±	0.05	0.76	±	0.06
Lymphocytes	×10^3^/μL	10.37	±	0.60	15.76	±	1.03**
Monocytes	×10^3^/μL	0.15	±	0.01	0.43	±	0.02***
Eosinophils	×10^3^/μL	0.15	±	0.01	0.24	±	0.03**
Basophils	×10^3^/μL	0.02	±	0.01	0.07	±	0.01**
Thrombocytes	×10^9^/L	795.30	±	11.70	730.40	±	33.33*
Plasma protein	g/L	70.00	±	7.94	63.33	±	4.16
I.I	unit	2.00	±	0.00	2.00	±	0.00

*n* = 3 rats per group

*Significant (*p* < 0.05); **significant (*p* < 0.01); ***significant (*p* < 0.001)

#### Histopathology

**Fig. 5:**
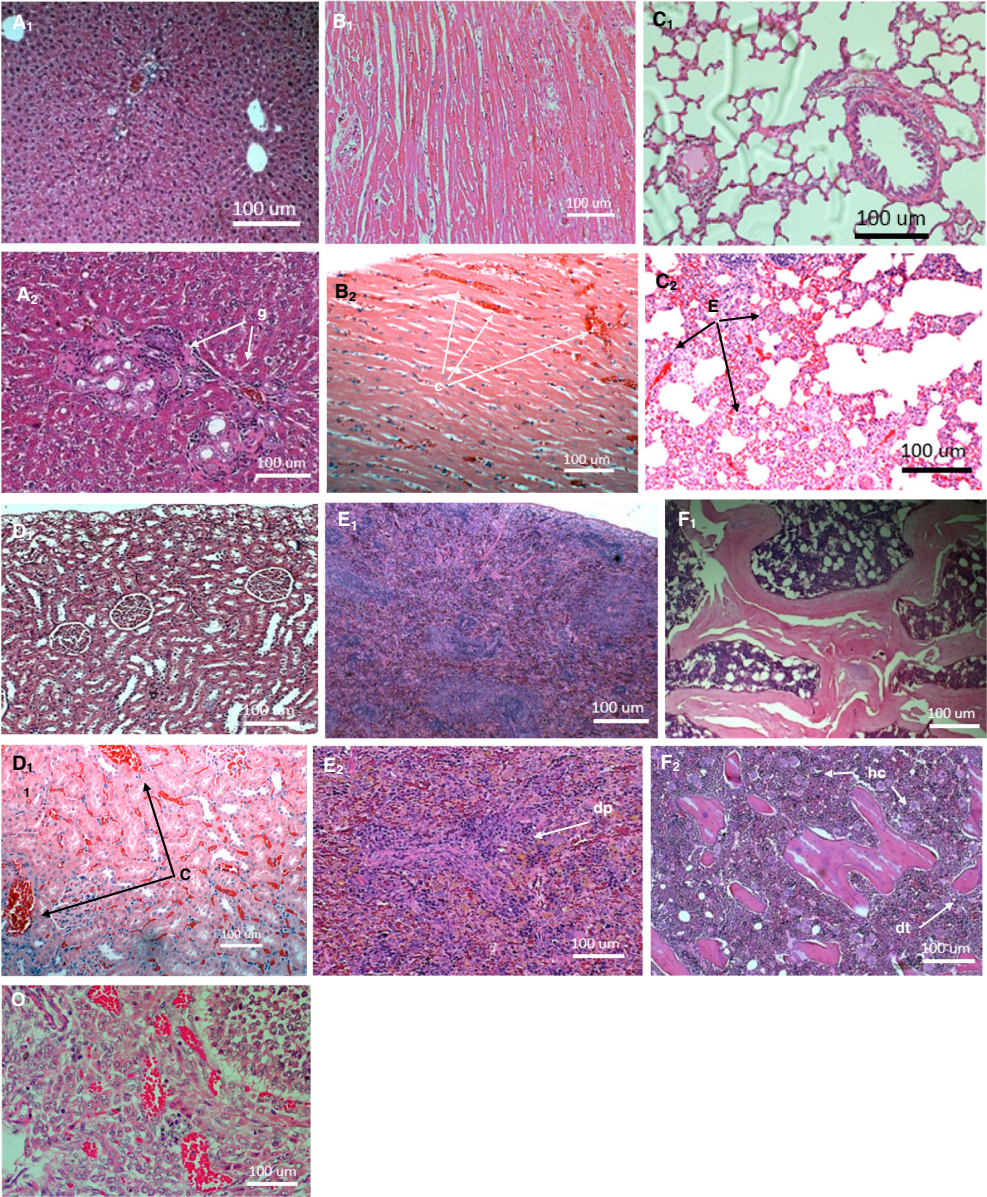
Micrographs of the normal and pathological liver (**a**), heart (**b**), lungs (**c**), kidney (**d**), spleen (**e**) and bone (**f**) of SD rats from 14-day acute toxicity of subcutaneous injections of 29,500 mg/m^2^ ANC. *Subscript 1* denotes normal tissues organ while *subscript 2* denotes pathological tissues. Note the granular formation (*g*) of the liver tissue; congested tissues; polymorphonuclear (PMN) cells infiltration (inflammation) and epithelialization of alveolar (*E*) septae of the lung tissue; and depleted white pulp (*dp*) of the splenic tissue and hypercellularity (*hc*) and trabecular degeneration (*dt*) of the bone tissue. The ovarian tissue (*O*) was observed to be normal

There were granular lesions in the liver, congestion of the heart and the kidneys and polymorphonuclear cell infiltration-associated thickening of alveolar septae in the lungs, pockets of white pulp depletion in the spleen and hypercellularity and cortical and trabecular degenerations in the tibial bone (Fig. 5). The ovaries and cutaneous tissues away from the site of injections were, however, observed to be normal.

### Repeated dose 28-day trial

#### Clinical observations and ophthalmology

**Fig. 6:**
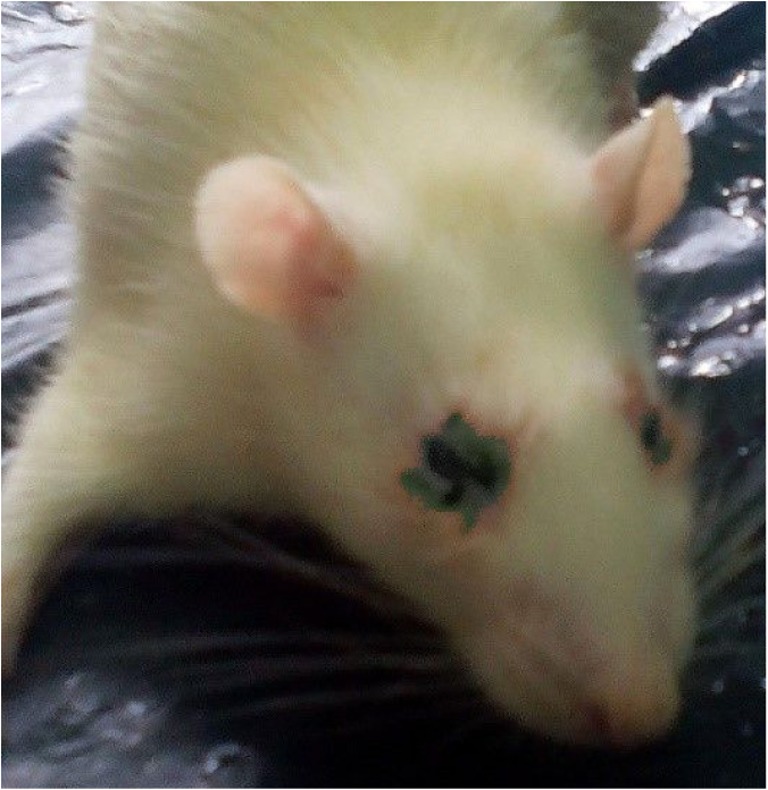
A SD rat from the repeated dose 28-day trial of ANC. Note the necrosis halo around the eye

No mortality was recorded at the end of the experiment. However, rats in the toxicity groups showed a dose dependent toxicity signs. There were signs of anorexia, roughened hair coat, lethargy and dyspnoea as the experiment advances. There were also fever and tachycardia and toxic signs of the eyes, characterized by necrotic lesions of the cornea and conjunctivae. The necrosis resulted in halos around both eyes (Fig. 6).

#### Evaluation of organ to body weight ratios from repeated dose 28-day trial of cockle shell-derived aragonite calcium carbonate nanocrystals

Tables 3 and 4 show the ratios of body and organ weights of SD rats in subchronic toxicity studies, respectively. There was a significant splenomegaly (*p* < 0.001) and significant decreased (*p* < 0.05) in lungs weight in the male of high toxicity (5900 mg/m^2^) group (Table 4).

**Table 3:** Body weights (g) of SD rats in repeated dose 28-day trial of cockle shell-derived aragonite calcium carbonate nanocrystals in SD rats (mean ± SD)

Group	Female			Male		
	Mean		SD	Mean		SD
Baseline end of experiment	202.83	±	7.41	242.83	±	9.21
0 mg/m^2^ (control)	240.17	±	6.31	351.00	±	5.97
59 mg/m^2^	241.00	±	4.34	350.33	±	7.69
590 mg/m^2^	232.83	±	3.31	339.17	±	4.07
5900 mg/m^2^	228.33	±	3.78	333.33	±	10.50

*n* = 6 per group

**Table 4:** Ratios of body and organ weights (g) from repeated dose 28-day trial on cockle shell-derived aragonite calcium carbonate nanocrystals in SD rats (mean ± SD)

Organ	0 mg/m^2^ (control).		SD	59 mg/m^2^		SD	590 mg/m^2^		SD	5900 mg/m^2^.		SD
Female												
Body weights	240.17	±	6.31	241.00	±	4.3	232.83	±	3.31	228.33	±	3.78
Liver	10.54	±	0.58	10.20	±	0.77	10.06	±	1.12	12.14	±	3.36
Spleen	0.85	±	0.05	1.02	±	0.04	1.15	±	0.47	1.70	±	1.01
Heart	1.04	±	0.04	1.02	±	0.05	1.01	±	0.05	1.05	±	0.08
Lungs	2.83	±	0.98	2.33	±	0.52	2.00	±	0.63	2.50	±	0.55
Kidney												
Left	1.05	±	0.08	1.03	±	0.07	1.02	±	0.13	1.18	±	0.40
Right	1.02	±	0.04	1.00	±	0.00	1.01	±	0.07	1.19	±	0.40
Male												
Body weight	351.00	±	5.97	350.33	±	7.69	339.17	±	4.07	333.33	±	10.5
Liver	12.17	±	1.33	12.72	±	2.57	14.51	±	1.37	14.93	±	2.07
Spleen	1.07	±	0.13	0.95	±	0.20	1.25	±	0.24	2.17	±	0.67**
Heart	1.03	±	0.07	1.09	±	0.10	1.21	±	0.15	1.59	±	0.51
Lungs	2.50	±	0.56	3.02	±	0.76	3.23	±	1.12	3.98	±	0.75*
Kidney												
Left	1.54	±	0.54	1.53	±	0.36	1.80	±	0.50	2.06	±	0.15
Right	1.32	±	0.37	1.64	±	0.40	1.62	±	0.46	1.97	±	0.33

*n* = 6 per group

*Significant (*p* < 0.05); **significant (*p* < 0.01)

#### Haematology and serum biochemistry

The serum biochemistry of the female medium (590 mg/m^2^) and high (5900 mg/m^2^) toxicity groups showed significant increases (*p* < 0.001) in ALT, ALP and AST levels, associated with liver diseases. There seems to be a negligent effects of the medium and high dosages on serum creatinine levels, as creatinine and urea of the male and female rats stabilized between these levels, while only the male showed stabilized urea at these levels (Tables 5 and 6). The medium- and high-dose levels of exposure were also observed to be associated with liver diseases in male rats, to a lesser extent (Table 6). The low toxicity (59 mg/m^2^) groups of both sexes showed no significant difference (*p* > 0.05) in levels of serum biochemical parameters studied (Tables 5 and 6).

**Table 5:** Blood results of female SD rats in the repeated dose 28-day trial of subcutaneous injection of ANC (mean ± SD)

Parameter	Unit	0 mg/m^2^ (control)		SD	59 mg/m^2^		SD	590 mg/m^2^		SD	5900 mg/m^2^		SD
Calcium	mmol/L	2.75	±	0.05	2.76	±	0.14	2.41	±	0.13***	2.45	±	0.08***
Alanine aminotransferase	U/L	22.55	±	3.22	28.16	±	7.90	40.98	±	5.12***	45.24	±	7.16***
Alkaline phosphatase	U/L	172.11	±	16.04	184.33	±	16.00	449.22	±	18.37***	659.33	±	18.11***
Aspartate aminotransferase	U/L	55.82	±	1.57	54.95	±	2.85	73.54	±	4.34***	132.35	±	9.21***
Creatine kinase	IU	264.07	±	21.76	266.17	±	38.00	311.55	±	40.22	321.34	±	47.02
Creatinine	umol/L	33.67	±	3.45	32.52	±	5.12	49.56	±	4.93***	65.66	±	2.79***
Phosphate	mmol/L	2.36.	±	0.21	2.54	±	0.17	2.55	±	0.22	2.57	±	0.38
Urea	mmol/L	13.98	±	0.88	12.83	±	0.71	16.12	±	1.62**	27.61	±	0.55***
Total protein	g/L	61.30	±	3.98	62.22	±	3.89	70.66	±	3.43**	74.87	±	6.73***
Lactate dehydrogenase	U/L	307.33	±	37.29	359.28	±	50.82	341.23	±	36.73	337.52	±	22.39
Red blood cells	×10^12^/L	8.29	±	0.36	8.56	±	0.63	7.21	±	0.35*	6.49	±	0.85***
Haemoglobin	g/L	151.3	±	7.45	154.87	±	5.84	140.15	±	9.70	139.66	±	5.66*
Haematocrit	L/L	0.47	±	0.03	0.44	±	0.09	0.37	±	0.02*	0.36	±	0.05*
MCV	fL	56.17	±	1.33	55.03	±	1.00	55.06	±	1.27	57.06	±	3 .00
MCHC	g/L	355.78	±	3.32	362.03	±	21.09	329	±	2.76*	315.89	±	19 .00***
White blood cells	×10^9^/L	5.22	±	0.48	6.37	±	4.36	8.97	±	1.08	14.68	±	1.24
Neutrophils	×10^3^/μL	0.70	±	0.04	0.68	±	0.03	0.73	±	0.03	0.78	±	0.02**
Lymphocytes	×10^3^/μL	10.82	±	0.23	10.83	±	0.29	13.74	±	0.16***^0^	15.67	±	0.35***
Monocytes	×10^3^/μL	0.14	±	0.02	0.17	±	0.03	0.19	±	0.02*	0.20	±	0.04**
Eosinophils	×10^3^/μL	0.16	±	0.03	0.15	±	0.02	0.22**	±	0.01	0.25	±	0.03***
Basophils	×10^3^/μL	0.00	±	0.00	0.00	±	0.00	0.00	±	0.00	0.00	±	0.00
Thrombocytes	×10^9^/L	594.81	±	17.13	582.13	±	21.61	439.66	±	14.80***	373.63	±	23.11***
Plasma protein	g/L	77.00	±	2.07	76.17	±	2.76	74.02	±	3.88	60.83	±	12.67**

*n* = 6 rats/group

*Significant (*p* < 0.05); **significant (*p* < 0.01); ***significant (*p* < 0.001)

**Table 6:** Blood results of male SD rats in the repeat dose 28-day trial of subcutaneous injection of ANC **(**Mean ± SD)

Parameter	Unit	0 mg/m^2^ (control)		SD	59 mg/m^2^		SD	590 mg/m^2^.		SD	5900 mg/m^2^		SD
Calcium	mmol/L	2.91	±	0.19	2.90	±	0.06	2.59	±	0.14*	2.49	±	0.34**
Alanine aminotransferase	U/L	21.48	±	8.90	24.20	±	4.07	33.68	±	3.71*	57.35	±	6.75***
Alkaline phosphatase	U/L	275.00	±	24.73	289.50	±	20.03	381.17	±	19.71***	760.52	±	10.09***
Aspartate aminotransferase	U/L	56.20	±	6.43	64.85	±	7.33	74.28	±	6.18**	133.73	±	9.10***
Creatine kinase	U/L	324	±	52.24	326.67	±	37.74	368.67	±	47.57	377.17	±	20.00
Creatinine	umol/L	33.00	±	1.03	35.00	±	2.76	65.83	±	2.85***	62.83	±	3.08***
Phosphate	mmol/L	2.40	±	0.28	2.35	±	0.10	2.74	±	0.20	2.75	±	0.4
Urea	mmol/L	14.90	±	0.90	14.18	±	0.72	18.93	±	0.92***	23.72	±	0.81***
Total protein	g/L	63.80	±	3.40	62.28	±	1.60	70.78	±	3.02*	71.75	±	6.12*
Lactate dehydrogenase	U/L	356.00	±	79.61	423.00	±	86.42	327.67	±	97.55	471.00	±	51.00
Red blood cells	×10^12^/L	8.89	±	0.44	8.85	±	0.49	7.84	±	0.86**	7.65	±	0.38*
Haemoglobin	g/L	164.97	±	8.76	167.17	±	6.52	151.12	±	6.16**	140.78	±	4.91***
Haematocrit	L/L	0.49	±	0.03	0.50	±	0.02	0.43	±	0.06	0.41	±	0.05*
MCV	fL	56.91	±	1.76	56.82	±	2.41	52.98	±	1.47*	53.05	±	3.03*
MCHC	g/L	342.00	±	5.57	331.04	±	11.11	339.61	±	7.58*	314.94	±	5.10***
White blood cells	×10^9^/L	13.31	±	0.73	14.17	±	1.13	15.25	±	1.12**	17.35	±	0.26***
Neutrophils	×10^3^/μL	0.94	±	0.05	0.98	±	0.02	0.96	±	0.05	1.01	±	0.05
Lymphocytes	×10^3^/μL	10.73	±	0.51	10.81	±	0.45	11.78	±	0.64*	13.99	±	0.55***
Monocytes	×10^3^/μL	0.23	±	0.05	0.22	±	0.03	0.32	±	0.02**	0.44	±	0.06***
Eosinophils	×10^3^/μL	0.12	±	0.00	0.13	±	0.01	0.17	±	0.01***	0.20	±	0.02***
Basophils	×10^3^/μL	0.00	±	0.00	0.00	±	0.00	0.00	±	0.00	0.00	±	0.00
Thrombocytes	×10^9^/L	1050.00	±	52.48	1041.83	±	46.92	944.33	±	39.24**	834.65	±	27.99***
Plasma protein	g/L	72.11	±	2.42	70.19	±	1.79	73.57	±	2.17	74.33	±	3.28

*n* = 6 rats/group

*Significant (*p* < 0.05); **significant (*p* < 0.01); ***significant (*p* < 0.001)

The haemogram of the female medium (590 mg/m^2^ body weight) and high (5900 mg/m^2^) toxicity groups showed significant decreases (*p* < 0.05 and 0.001) in red blood cells, haemoglobin, haematocrit and mean corpuscular haemoglobin concentration (MCHC). The two dosages were also associated with blood thinning characterized by thrombocytopenia (*p* < 0.001) (Table 5). Similar trends were observed in the male medium (590 mg/m^2^ body weight) and high (5900 mg/m^2^) toxicity groups of this study (Table 6). Significant eosinophilia (*p* < 0.001) was also recorded in the female high (5900 mg/m^2^) toxicity and the male medium (590 mg/m^2^ body weight) and high (5900 mg/m^2^) toxicity (*p* < 0.05 and 0.001) groups. The low toxicity groups (59 mg/m^2^) of both sexes showed no significant changes (*p* > 0.05) in levels of the haemogram parameters studied (Tables 5 and 6).

#### Gross lesions and histopathology

**Fig. 7:**
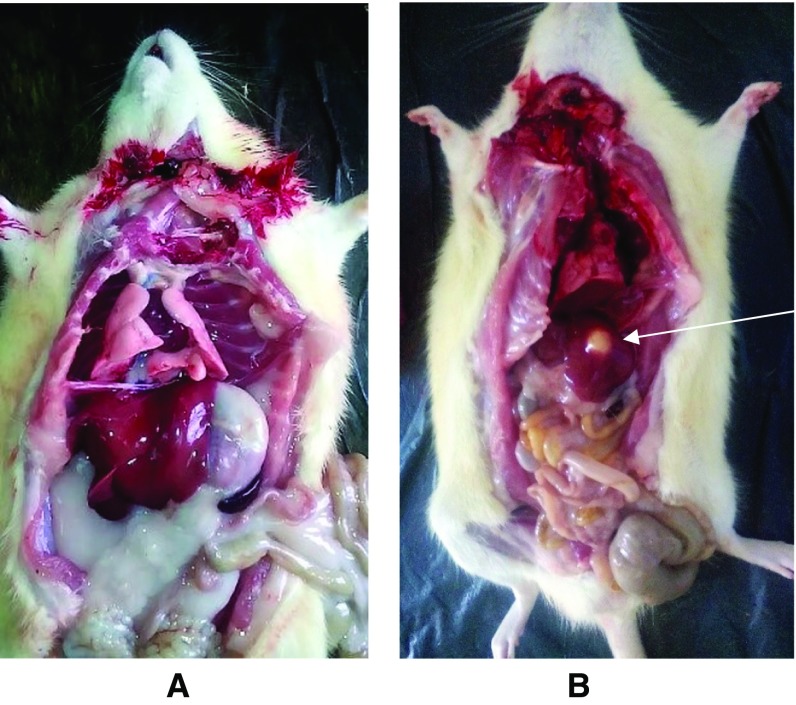
Photographs of SD rats from control (**a**) and high toxicity (5900 mg/m^2^) (**b**) groups depicting a normal and a liver with abscess in **a** and **b**, respectively. Note the centrally located circumscribed abscess (*arrow*) in the liver of rat from the high toxicity group (**b**)

There were marked splenomegaly and hepatomegaly in the high toxicity group of both sexes. The hepatomegaly was also associated with fatty infiltration (Fig. 7). The severity of the histopathological lesions in the viscera and bones of the toxicity groups in both sexes were dosage specific, while the gonads showed no significant lesion.

**Fig. 8:**
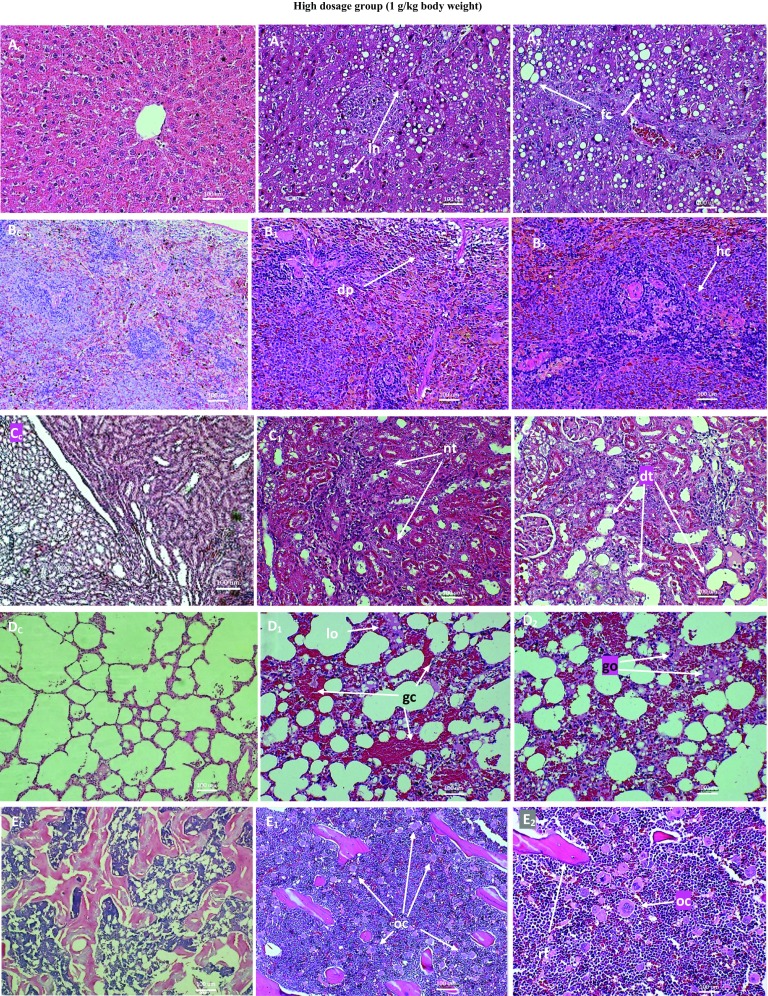
Micrographs of the normal and pathological liver (**a**), spleen (**b**), kidney (**c**), lung (**d**) and bone (**e**) of SD rats from high dosage (5900 mg/m^2^) group of 28-day subchronic toxicity of subcutaneous ANC injections. *Subscript C* denotes normal tissues while *subscripts 1* and *2* denote pathological tissues. Note the multifoci necrosis (*ln*, blackish) and marked fatty cells (*fc*, whitish) lodgement in the liver tissue; the depopulation of the white pulp (*dp*) and hypercellularity (*hc*) of the splenic tissue; the degenerations (*dt*) and necrosis (*nt*) of renal tubules with mild glomerular lesion; the generalized congestion (*gc*) and oedema (*go*) of the lung tissue; and the marked increase in osteoclast population (*oc*) and trabecular resorption (*rt*) in the bone tissue

Figure 8 shows lesions, as against normal tissues, from high dosage group of 28-day subchronic toxicity of ANC in SD rats. There were marked fatty cells in the liver due to the inability of the liver to metabolize fats, characteristic of liver damage. The spleen showed no distinction between red pulp and white pulp due to depopulation of the white pulp and hypercellularity. There were macrophages and giant cells infiltration. The kidneys showed multifocal interstitial polymorphonuclear infiltration. The glomeruli showed mild abnormalities. There were vacuolar degenerations and marked degenerations and necrosis of renal tubules. There were generalized congestion and exudates in the lungs. The proximal tibial extremity showed hypercellularity and marked increase in osteoclast population, without commensurate increase in osteoblast population. This led to an attendant marked trabecular and cortical resorption. No significant lesions were associated with the heart and gonads.

**Fig. 9:**
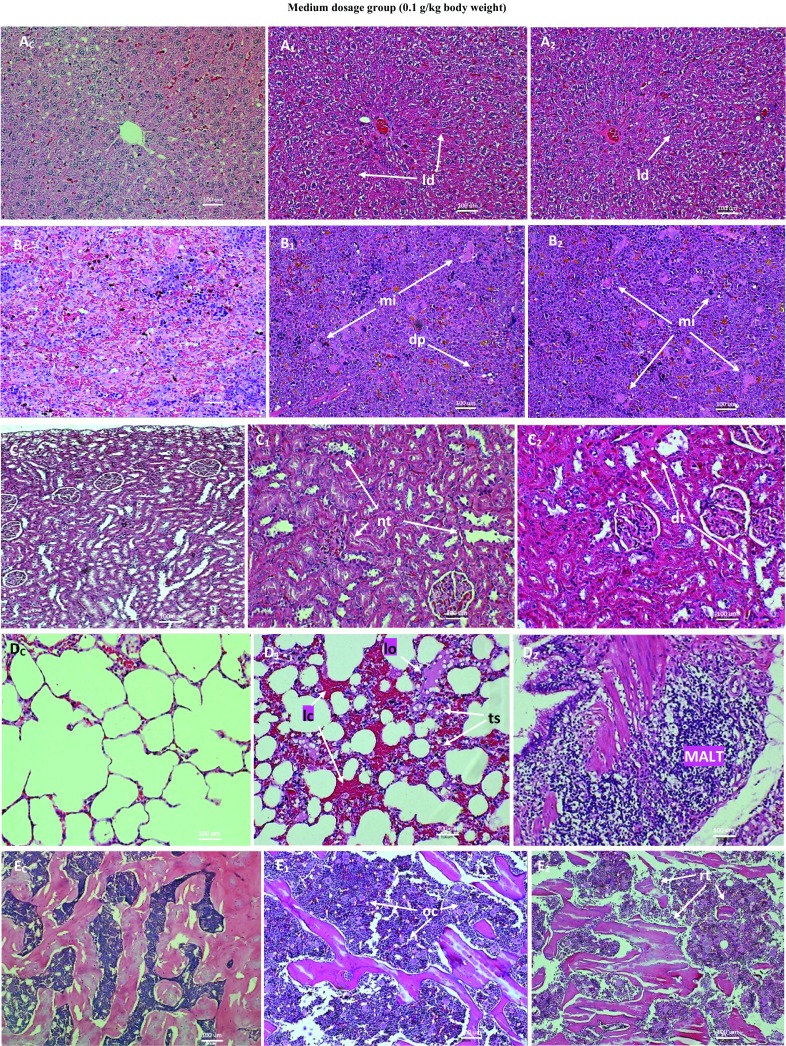
Micrographs of the normal and pathological liver (**a**), spleen (**b**), kidney (**c**), lung (**d**) and bone (E) of SD rats from medium dosage (590 mg/m^2^) group of 28-day subchronic toxicity of subcutaneous ANC injections. *Subscript C* denotes normal tissues while *subscripts 1* and *2* denote pathological tissues. Note the mild lobar degenerations without necrosis (*ld*) in the liver tissue; the macrophage infiltration (*mi*) and depletion of white pulp (*dp*) areas in the splenic tissue; the mild glomerular and tubular degeneration (*dt*) and necrosis in tubules (*nt*) of the kidney tissue; the congestion (*lc*), oedema (*lo*), thickened alveolar septae (*ts*) and the mild proliferation of mucosa-associated lymphoid tissue (MALT) cells in the lung tissue; and the moderate increase in osteoclast population and trabecular resorption in the bone tissue

Figure 9 shows lesions, as against normal tissues, from medium dosage group of repeated dose 28-day trial of ANC in SD rats. The liver showed generalized mild degeneration without necrosis. The spleen showed large areas of macrophage infiltration and depletion of white pulp areas. There were mild multifocal tubular degeneration and necrosis in the kidneys. The glomeruli showed mild abnormalities. The lungs showed localized congestion and exudation with attendant increase in thickness of the alveolar septae due to elicited interstitial infiltration. There was also mild proliferation of mucosa-associated lymphoid tissue (MALT) cells. The proximal tibial extremity showed hypercellularity, moderate increase in osteoclast population and associated trabecular and cortical resorption. There were no significant lesions in the heart and gonads.

**Fig. 10:**
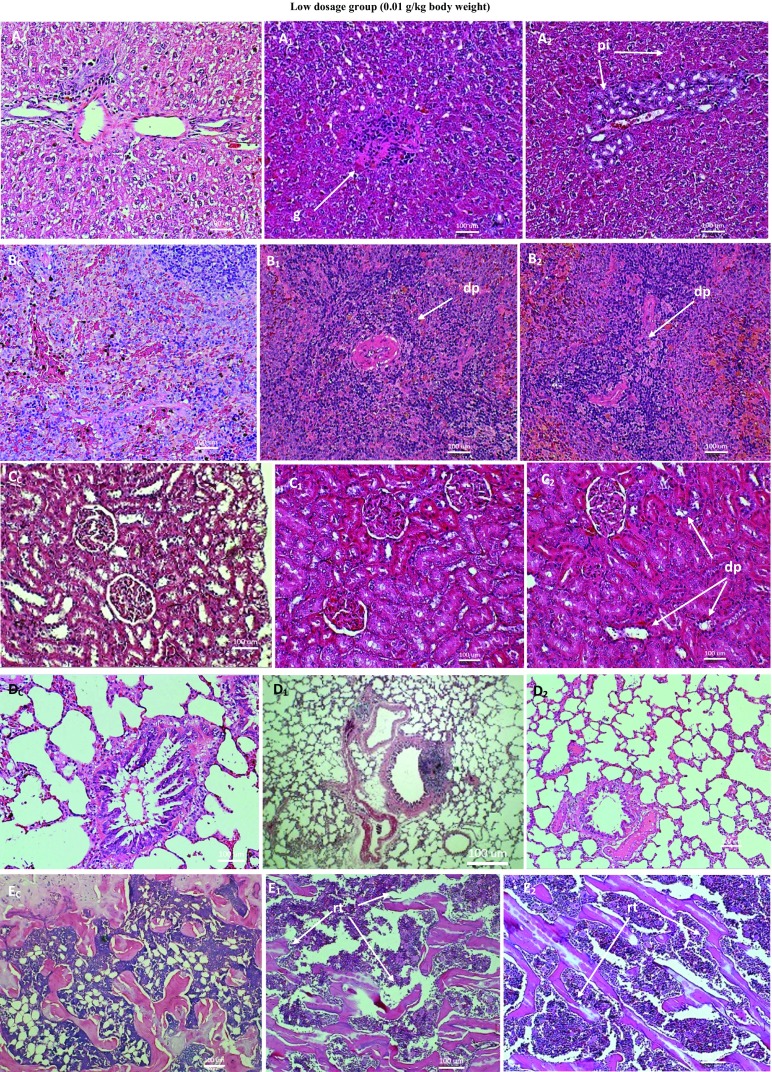
Micrographs of the normal and pathological liver (**a**), spleen (**b**), kidney (**c**), lung (**d**) and bone (**e**) of SD rats from low dosage (59 mg/m^2^) group of 28-day subchronic toxicity of subcutaneous ANC injections. *Subscript C* denotes normal tissues organ while *subscripts 1* and *2* denote pathological tissues. Note the granular (*g*) formation and periportal infiltration (*pi*) in the liver tissue; mild depletion of white pulp (*dp*) in the splenic tissue; the reversible degenerations without necrosis of renal tubules (*dt*); and the mild trabecular destruction (*rt*) in the bone tissue. The lung tissues show no significant lesion

Figure 10 shows lesions, as against normal tissues, from low dosage group of repeated dose 28-day trial of ANC in SD rats. There was granulation of the liver due to the efforts of macrophages in curtailing the spread of ANC to the liver. There were also areas of periportal infiltration of polymorphonuclear cells. There were pockets of white pulp depletion in the spleen. There was generalized mild degeneration without necrosis of renal tubules. There was mild trabecular destruction and normal cortical thickness in the proximal tibial extremity. The lungs, the heart and the gonads showed no significant lesion.

## Discussion

Physicochemical characterization of NPs has been identified as the first step towards meaningful understanding of its in vitro or in vivo biological data or inter-laboratory comparison (McNeil 2011). Detailed physicochemical characterization and cytocompatibility of ANC had earlier been determined (Jaji et al. 2017).

Cockle shells are remarkable sources of naturally purified aragonite polymorphs of calcium carbonate (Islam 2012). Though calcium carbonate is renowned for its multifaceted applicability in fields of science (Epple 2003; Manolova et al. 2008; Colfen and Mann 2003; McLeod et al. 2004; Yu et al. 2006; Naka et al. 2006; Islam et al. 2011), it is poised for greater advancements in nanomedicine. Nanomedicine offers many prospects and benefits to medical research by making pharmaceuticals more efficacious (McNeil 2011). The nanotechnological top-down method of synthesis of ANC from this natural reservoir holds huge benefits. It enables obtaining aragonite crystals in their natural forms while retain most of their special features (Islam et al. 2011, 2012). The method enabled synthesis of spherical ANC of 30 ± 5 nm size, as determined by TEM and FESEM. Although, morphology, structure, size, surface area oil adsorption and chemical purity are important determinant factors for the use of calcium carbonate in varying applications, morphology appears to be the most important. As such, synthesis of calcium carbonate crystals with homogenous shape and size is now a topic of research due to the interesting mechanical and optical properties (Loy et al. 2004; Xu et al. 2006; Zhang et al. 2008; Zhanga et al. 2010).

The use of inorganic NPs as drug delivery carriers has gained wide concerns (Zhanga et al. 2010). Though, calcium carbonate NPs have shown promising potential for the development of carriers for drugs and are gaining recognitions as successful nanocarriers for subcutaneous delivery of biologicals (Ueno et al. 2004; He et al. 2008; Higaki et al. 2006; Zhang et al. 2012). There is paucity of research on their safe dosage for maximizing their therapeutic activity without harming biosystems (Zhang et al. 2012). This study demonstrates the safety of ANC as a potential agent for subcutaneous delivery of biologics and drugs. No mortality was recorded at the end of the acute and subchronic toxicity experiments. With a LD_50_ of 6450 mg/kg body weight (body weight), calcium carbonate has a wide margin of safety and low acute toxicity (Aguilar et al. 2011). There was no sign of toxicity from the initial dosages used in the acute toxicity experiment (single doses of 1770 and 11,800 mg/m^2^). However, the final dosage (29,500 mg/m^2^) that was eventually used was for the 14-day acute toxicity study was associated with some toxic signs and lesions. The eventual recourse to high-dose usage was in line with the Organization for Economic Cooperation and Development (OECD) laid guidelines preliminary trial doses for acute toxicity studies, test number 423 (OECD 2010).

Moderate histopathological lesions were observed in rats of the 14-day acute toxicity group. To the best of the knowledge of the authors of this recent study, no record is available detailing the toxicity of subcutaneous administration of calcium carbonate. Available data on calcium carbonate toxicity are related to mild to moderate oral toxicity from its usage as mineral supplement. This may not be unconnected to the fact that calcium carbonate from oral route dissociates into its constituent ions in the acid milieu of the stomach. Some of the component calcium is absorbed, via active transport or passive diffusion (Aguilar et al. 2011), while the greater percentage of the unabsorbed calcium is complexed to bile acids, free fatty acids and oxalic acid and excreted with the faeces (Heaney 2002). More so, most of the available toxicological data on calcium carbonate were on the micron, other than the nano sized. Toxicological findings have revealed high toxicity of NPs compared to micron sized particles of the same composition, thus, posing questions on their human health importance (Karlsson et al. 2009).

There was marked splenomegaly and hepatomegaly in the high toxicity group of both sexes. The hepatomegaly was also associated with fatty degeneration (lipidosis). Lu et al. (2010) reported mild splenomegaly and hepatomegaly associated with mesoporous silica NPs for cancer therapy in mice. In a study on 7-day acute toxicity of single oral dose, 11,770 mg/m^2^ gavage of nano versus micron calcium carbonate and 261 U vitamin D3/kg body weight in mice, no mortality or changes related to treatment were recorded in either group (Huang et al. 2009). Similarly, an acute toxicity study recorded no treatment-related effects in female Sprague-Dawley rats administered a single dose of 2000 mg calcium carbonate/kg body weight by gavage (SafePharma 2008).

Though calcium carbonate does not meet the criteria for classification as dangerous substances according to Directive 67/548/EEC as amended on eye irritation due to topical calcium carbonate (CCA 2005), the present study recorded necrotizing lesion around the eyelids of rats from the high toxicity group following the 28-day subchronic toxicity study. This could be due to the systemic effect of ANC following the subcutaneous route of administration. Dendritic cells are the first line of contact following subcutaneous administration of immunogenic nanoparticle compounds; these cells engulf the foreign material based on its size and present to antigen presenting cells, which move to resident reticuloendothelial tissues such as the spleen, tonsils and lymph nodes through the lymphatic drainage systems. In the study, large particulate materials (500–1000 nm) were mostly found in DC at the injection site, while small (20–200 nm) were found in DC of close by lymph nodes (Manolova et al. 2008).

Most of the haematological parameters in the repeated dose 28-day trial groups showed no significant variations. The changes in haematological parameters recorded from this study are related to cascade of immunological responses caused by ANC. An earlier study by Harlan Laboratories (2010), on a combined repeat dose oral toxicity/reproduction/developmental toxicity screening study with “nano” calcium carbonate in Wistar rats (Han™/HsdRccHan™/WIST strain), had documented minor haematological changes in males receiving 5900 mg/m^2^/day. NPs have been associated with changes in haematological parameters (Smith et al. 2007; Xie et al. 2011; Khabbazi et al. 2015).

The severity of the histopathological lesions in the viscera and bones of the toxicity groups in both sexes were dosage dependent and not in agreement with earlier reports by Harlan Laboratories (2010) and Aguilar et al. (2011) that documented the absence of treatment-related effects from studies on oral administration of calcium carbonate. This was attributed to the difference in route of administration of calcium carbonate in both studies and size in the later study, as discussed above. The serious toxicity lesions were limited to the medium to high subcutaneous dose groups of ANC. The gonads and skin showed no significant lesion. It has been observed that apart from blood, phagocytosis and endocytosis of NPs by body cells have also been observed to play very important roles in their further spread to distant organs (Garnett and Kallinteri 2006; Yacobi et al. 2010; Greulich et al. 2011). Irrespective of natural barriers, low concentrations of NPs have been found in the liver, the spleen, the heart and the brain (Ji et al. 2006; Oberdorster et al. 2002).

ANC at low dosage is largely a safe inorganic crystal with potentials for subcutaneous delivery of biologicals and drugs, as well as a calcium carbonate supplement. The low dosage (59 mg/m^2^ body weight) group showed the high safety margin of ANC. The study was able to correct the impression that CaCO_3_ is generally safe. The safety of CaCO_3_ nanocrystals in vivo is dependent on its concentration and route of administration.
